# Melatonin Supplementation in Patients with Complete Tetraplegia and Poor Sleep

**DOI:** 10.1155/2013/128197

**Published:** 2013-03-12

**Authors:** Jo Spong, Gerard A. Kennedy, Douglas J. Brown, Stuart M. Armstrong, David J. Berlowitz

**Affiliations:** ^1^Institute for Breathing and Sleep, Austin Hospital, Heidelberg, VIC 3084, Australia; ^2^School of Social Sciences & Psychology, Victoria University, St. Albans, VIC 3021, Australia; ^3^Victorian Spinal Cord Service, Austin Hospital, Heidelberg, VIC 3084, Australia; ^4^Brain Sciences Institute, Swinburne University, Hawthorn, VIC 3122, Australia; ^5^The Bronowski Institute of Behavioural Neuroscience, Kyneton, VIC 3444, Australia

## Abstract

People with complete tetraplegia have interrupted melatonin production and commonly report poor sleep. Whether the two are related is unclear. This pilot study investigated whether nightly supplementation of 3 mg melatonin would improve objective and subjective sleep in tetraplegia. Five participants with motor and sensory complete tetraplegia ingested 3 mg melatonin (capsule) two hours prior to usual sleep time for two weeks. Full portable sleep studies were conducted in participants' homes on the night before commencing melatonin supplementation (baseline) and on the last night of the supplementation period. Endogenous melatonin levels were determined by assaying saliva samples collected the night of (just prior to sleep) and morning after (upon awakening) each sleep study. Prior to each sleep study measures of state sleepiness and sleep behaviour were collected. The results showed that 3 mg of melatonin increased salivary melatonin from near zero levels at baseline in all but one participant. A delay in time to Rapid Eye Movement sleep, and an increase in stage 2 sleep were observed along with improved subjective sleep experience with a reduction in time to fall asleep, improved quality of sleep and fewer awakenings during the night reported. Daytime sleepiness increased however. A randomised, placebo controlled trial with a larger sample is required to further explore and confirm these findings.

## 1. Introduction

For people living with tetraplegia, excessive daytime sleepiness, disturbed and poor quality sleep are a common problem [1]. A number of factors contribute to disturbed sleep in people with tetraplegia with the absence of increase in evening endogenous melatonin production after a complete cervical spinal cord injury (SCI) [2, 3] potentially being one. Melatonin is secreted nocturnally by the pineal gland and is believed to play a major modulatory role in the timing of circadian rhythms including the sleep-wake cycle [4]. The daily rhythm of melatonin secretion is regulated by an endogenous pacemaker, the suprachiasmatic nucleus (SCN, “circadian clock”) which signals the pineal gland via a circuitous route involving other hypothalamic nuclei, brain stem nuclei, the spinal cord, and peripheral sympathetic neurons from the superior cervical ganglion (SCG) [4]. Melatonin levels typically begin to increase two to three hours before sleep with peak levels between 02:00 and 04:00 and trough levels during the day [4].

Melatonin secretion following a SCI is low or abolished in those with complete tetraplegia, but relatively normal in those with complete paraplegia [2, 3, 5–7]. This suggests that the neural pathway controlling melatonin secretion passes through the cervical spinal cord and is interrupted between the SCN and the SCG. The fibres to the SCG are routed along with those of the autonomic nervous system and thus are disrupted in a manner analogous to the disruption that causes autonomic dysfunction [8]. Only one study has investigated the effect of abolished melatonin secretion on sleep in tetraplegia [9]. The study showed that total sleep time (TST) and sleep efficiency were significantly reduced, and rapid eye movement (REM) latency significantly delayed in complete tetraplegia compared with paraplegia. It was suggested that melatonin supplementation might assist in the restoration of normal sleep in people with tetraplegia [9]. 

Timed daily doses of melatonin in able-bodied people have been shown to reduce sleep onset time, improve sleep maintenance, increase REM sleep, and correct circadian based insomnias [10–15]. Timed daily administration of exogenous melatonin is also the current treatment of choice for “non-24-hour sleep/wake disorder” (free-running circadian rhythms) in people who are functionally blind and have complete or partial attenuation of ocular light transmission from the retina to the circadian clock [16, 17]. Despite such evidence that melatonin supplementation can improve sleep in able-bodied and blind people, there have been no studies investigating whether melatonin supplementation can improve sleep in people with complete tetraplegia. This pilot study is the first to investigate whether timed, nightly supplementation of 3 mg melatonin affects sleep in tetraplegia.

## 2. Methods

### 2.1. Participants

Five people (four male) with complete tetraplegia who had been treated by the Victorian Spinal Cord Service, Australia, were recruited. Patients diagnosed as having “complete” tetraplegia (American Spinal Injuries Association Impairment Scale (AIS) level A) have no motor or sensory function below the lesion level [18]. Two able-bodied participants were recruited to provide melatonin positive controls. Exclusion criteria were a history of major psychological/psychiatric disturbances, neurological damage (other than SCI), and significant comorbidity. Participants did not undertake transmeridian flight prior to or during the study. The project was approved by the Austin Health Human Research Ethics Committee, and all participants provided informed consent before the study began. The study is registered at http://www.anzctr.org.au/ (083167). 

### 2.2. Procedure

Participants completed a 7-day sleep diary recording their intake of medication, caffeine, and alcohol, the type and duration of exercise performed, their bedtime, sleep onset and awakening times, the number of nocturnal awakenings, and the duration of any daytime naps. At the completion of the 7-day sleep diary, participants underwent a baseline sleep study (polysomnography) recorded in their home using a portable sleep monitoring device (Compumedics Somte, Abbottsford, VIC, Australia). Sleep study measures included central electroencephalography (C4/A1, C3/A2), bilateral electrooculography, electromyography (chin and diaphragmatic), electrocardiography, blood oxygen saturation, nasal pressure (airflow), leg movements, body position, and respiratory movements of the chest and abdomen. The portable sleep monitoring device does not contain a light sensor and as such, sleep efficiency and sleep onset latency could not be measured.

Beginning on the evening following the baseline sleep study, participants ingested one 3 mg melatonin capsule every night for two weeks, approximately two hours before their usual sleep time; the typical, natural onset time of endogenous melatonin [19]. The dose of 3 mg of melatonin for supplementation reflected the typical dose of melatonin prescribed in Australia. Participants had standard home lighting which would not have interfered with their melatonin production. On the last night of the two-week melatonin supplementation period, participants underwent a second home sleep study. Sleep was staged in 30-second epochs, arousals marked, respiratory events scored, and summary indices calculated according to international standard criteria [20, 21] by an independent, experienced sleep scientist blinded to treatment phase and participant.

Prior to each sleep study participants completed two questionnaires, the Karolinska Sleepiness Scale (KSS) [22] and the Basic Nordic Sleepiness Questionnaire (BNSQ) [23]. The KSS measures state sleepiness on a 9-point scale where 1 indicates feeling extremely alert and 9 indicates extreme sleepiness. Participants were asked to rate their level of sleepiness at mid-day of the day of each sleep study. The KSS is a more appropriate measure of state sleepiness for this population compared to others which ask questions that are often not applicable to people with this level of disability. The KSS has also been used in previous research investigating sleep in people with tetraplegia [24, 25]. The BNSQ is a general sleep questionnaire that has been validated in the spinal patient population [26]. It assesses sleep behaviour and symptoms of sleep disturbances during the past three months [1, 26] via 14 five-point scale questions (high score indicates a poorer condition or greater frequency) and six quantitative questions. In this study, BNSQ questions 1, 2, and 7 were used to assess sleep initiation, questions 3, 4, and 5 to examine waking behavior during sleep and early morning, questions 8, 9, and 15 to assess sleepiness (typical trait morning and daytime sleepiness), questions 10 and 11 to assess daytime functioning (inability to maintain wakefulness), questions 6, 12, 13, 14, and 20 to assess sleep quality, and questions 16, 17, and 18 to assess the presence of Obstructive Sleep Apnoea (OSA). The BNSQ that was administered on the last night of melatonin supplementation assessed participants' sleep/wake behavior and symptoms of sleep disturbances across the previous two weeks (melatonin supplementation period).

Saliva samples (2–4 mL) were collected just prior to sleep onset on the night of each sleep study and the following morning upon awakening to measure the participants' endogenous melatonin levels. Saliva collected from the two able-bodied participants (melatonin controls) provided an assay reference level. The control participants did not perform any other task in this study.

### 2.3. Data Analysis

Results from the sleep diary, sleep studies, and questionnaires are summarized as medians (range) and Wilcoxon Signed Rank Test used to compare baseline and melatonin supplementation data. For the 7-day sleep diary, medians for each variable were calculated for each participant and then group medians generated from these values. Missing values on the questionnaire items were generated by calculating the sample average for the specific item. BNSQ items relating to work days were analysed for those known to be in the work force (*n* = 2). Similarly, BNSQ items relating to OSA were only analysed for those not being treated for OSA. The error range associated with the saliva assay of melatonin is 10.2% (E. Sorich, ARL Pathology, Personal Communication). The reportable concentration levels of the saliva assay ranged between 0.5 and 50 pg/mL. 50 pg/mL was the maximum dilution level for this saliva assay (J. Evans, Healthscope Pathology, Personal Communication).

## 3. Results

The median (range) age of the tetraplegia participants was 42 years (26–68 yrs), Body Mass Index (kg/m^2^) was 20.8 (18.5–32.9), and number of years living with complete tetraplegia was 17.1 years (4.1–23.9 yrs). At the time of the study, three participants had a diagnosis of OSA, two of whom were treated with Continuous Positive Airway Pressure (CPAP) therapy and one with a mandibular advancement splint. All participants continued their usual medications throughout the trial period. The individual characteristics of each participant are presented in Table 1. 

### 3.1. Sleep Diary

Median (range) analyses from the 7-day sleep diary showed that the usual bedtime of the sample was 22:00 h (21:00 h–00:00 h) and time to fall asleep was 15 minutes (1.5–180 mins). The median number of awakenings was one (0–3) where the group would be awake for approximately 15 mins (0 sec–120 mins) before falling back to sleep. Morning wake time was 06:30 h (06:00 h–06:30 h) with a rising out of bed at 07:15 h (06:00 h-08:30 h). The number of naps taken during the day ranged between zero and one. Four participants reported doing exercise (stretches, weights) during the week for between 30 and 120 minutes. Intake of alcohol, drugs, or caffeine during the week was reported by four participants.

### 3.2. Saliva Samples: Endogenous Melatonin Concentration Levels

**Figure 1:**
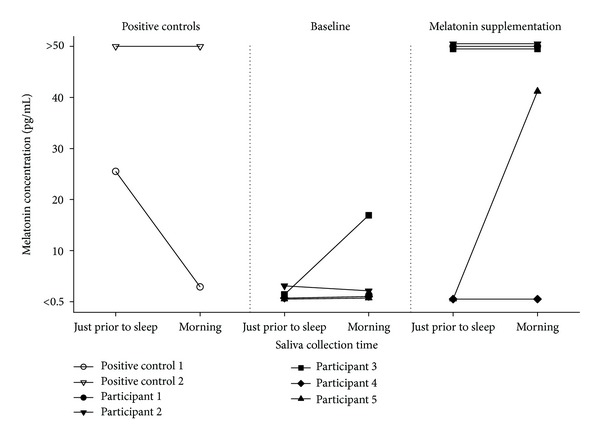
Melatonin concentrations (pg/mL) from saliva sampled just prior to sleep and after awakening for the positive controls and for the participants with complete tetraplegia at baseline and two weeks after melatonin supplementation. Note: reportable pg/mL range for this saliva assay was 0.5 to 50. Melatonin concentration values for three participants with a ceiling pg/ml were slightly altered on graph to show visually that three participants obtained this maximum reportable value.

Melatonin levels measured at baseline for the tetraplegia sample were essentially zero (Figure 1) in all but participant 3. Supplementation of 3 mg melatonin two hours prior to sleep increased melatonin concentration to levels comparable with those of the positive controls in all but participant 4 (Figure 1). 

### 3.3. Sleep Studies: Objective Sleep

**Figure 2:**
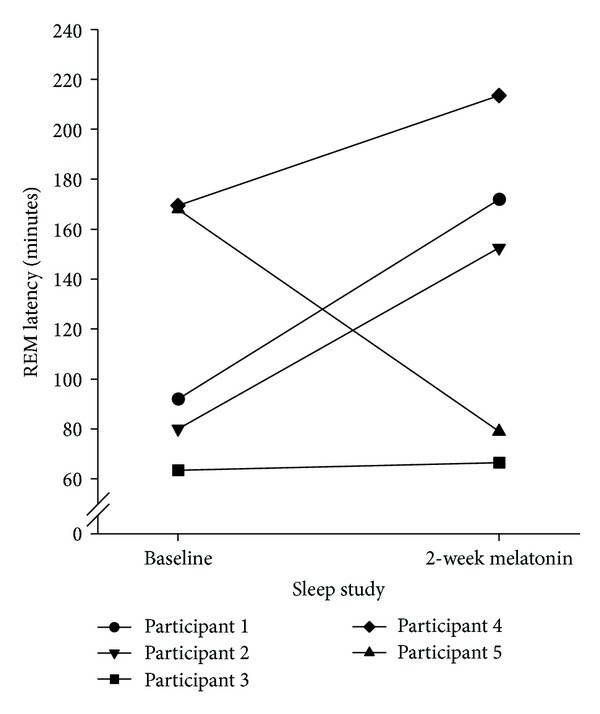
Individual REM latencies recorded for each participant at baseline (the night prior to commencing melatonin supplementation) and on last night of two-week melatonin supplementation period.

The sleep parameters showing a noticeable change following melatonin supplementation were the proportion of stage two sleep and REM latency. Administration of 3 mg melatonin resulted in a 15.4% increase in the proportion of time spent in stage 2 sleep and a one-hour delay in REM latency (Table 2). Examination of REM latency for each participant (Figure 2) shows that the delay was pronounced for three of the five participants. Conversely, REM latency shortened for participant 5. No statistically significant differences were observed in these trends.

### 3.4. Questionnaires: Subjective Sleep

**Figure 3:**
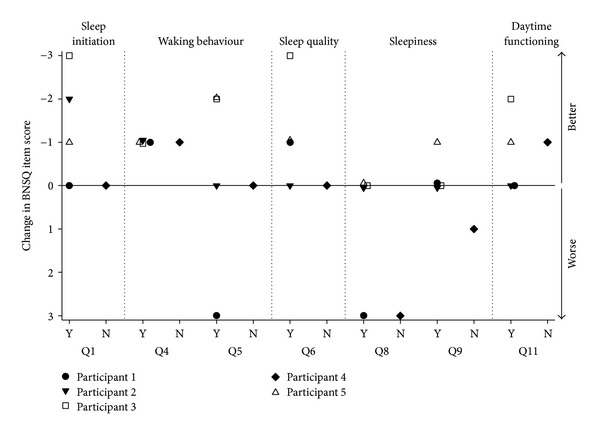
Change in individual participant responses to BNSQ scaled questions after two weeks of melatonin supplementation. Y = participants who showed an increase in melatonin concentration levels, N = the participant with no change in melatonin concentration level. Negative scores indicate improvement on the BNSQ item; positive scores indicate poorer sleep experience or functioning, 0 represents no change.

Subjective sleep improved following melatonin supplementation (Table 3), specifically for the BNSQ questions regarding sleep initiation (questions 1 and 2(b)), awakenings during the night and early morning (questions 4 and 5), quality of sleep (question 6), and daytime functioning (question 11); however, feeling sleepy during the day increased following melatonin supplementation (questions 8 and 9), as did sleep apnoea symptoms (questions 16 and 18). There was no change in state sleepiness (KSS, Table 3). Individual responses to the sleep-related scaled BNSQ questions showed that those with an increase in salivary melatonin generally reported improved sleep (Figure 3). Conversely, participant 4 who showed no change in salivary melatonin trended towards reporting similar or poorer rather than improved sleep. Only the change in BNSQ 4 following melatonin supplementation reached statistical significance.

## 4. Discussion

This study replicates earlier reports of reduced melatonin production in people with complete tetraplegia [2, 3]. It also shows that it is possible to increase circulating melatonin concentration levels in people with chronic complete tetraplegia by administering exogenous melatonin and that the increase in melatonin is generally associated with an improved subjective sleep experience. 

Although the melatonin concentration levels of the tetraplegia sample were generally low at baseline there was a degree of intersubject variability in our findings. The higher baseline melatonin reading in the morning for participant 3 was unexpected. Possible explanations include preservation of the melatonin pathway and assay variability. The increase in melatonin concentration following supplementation was also variable, with participant 4 showing no observable rise in melatonin at all and the increase in melatonin level for participant 5 only evident in the morning sample. This variability may possibly be due to differences in hepatic catabolism or other changes in metabolism in participants deprived of endogenous melatonin for many years. The results of participants 4 and 5 suggest poor or delayed absorption of melatonin. 

Benzodiazepine medication has been found to suppress the nocturnal rise in plasma melatonin or shift its day-night rhythmicity [27]. This drug interaction may also explain why participant 4, who was taking diazepam at the time of this study, had no observable rise in salivary melatonin; however, melatonin concentration increased for participant 3 who was also taking diazepam, suggesting that any drug interaction may have a variable consequence across individuals.

This study has shown that it is possible to increase circulating melatonin levels in people with chronic tetraplegia; however whether such levels are equivalent to or exceed normal nocturnal values is impossible to determine with the limited reportable concentration levels of this saliva assay (i.e., 0.5 to 50 pg/mL). 

Despite the increase in saliva melatonin concentration levels, melatonin supplementation did not improve TST or reduce REM latency, the sleep parameters identified in earlier research [9] as being significantly altered for people with complete tetraplegia. We observed that the TST remained relatively stable and REM latency was increased by a further 60 minutes. Previous authors have found similar prolongation of REM following the administration of one and five mg of melatonin to people with insomnia and healthy participants 15 minutes or two hours prior to bedtime [28, 29]. In both the previous research and the current study, the importance of and the mechanism underlying the delay in REM latency after melatonin supplementation is not clear. 

Benzodiazepine medications have been known to delay or suppress REM sleep [30]. Two participants in our trial were taking Diazepam and one taking Temazepam; however any affect on REM latency would be expected to be similar between these participant's baseline and melatonin supplementation sleep studies. Furthermore, an effect of medication does not account for the pronounced delay in REM latency for the participants who were not using benzodiazepines. 

The improvement in subjective sleep experience following melatonin supplementation appeared specific to sleep initiation, sleep quality, daytime functioning, and awakenings. There is no evidence to suggest that the improved subjective sleep was due to the chronobiotic (phase shifting) properties of melatonin [31]. Rather, participants may have experienced the hypnotic properties associated with melatonin. Exogenous melatonin may act as a hypnotic by attenuating wake-promoting signals from the SCN [32, 33] and/or as a hypothermic, decreasing body temperature to promote sleep [34, 35]. It is generally accepted that melatonin administered during the day, when endogenous levels are absent, has a sleep-promoting effect [13, 33, 36, 37]. Past research that has administered melatonin closer to the time of habitual sleep in able-bodied subjects, however, has produced less consistent findings [32, 38–40]. In the current study we added exogenous melatonin to a system with no endogenous melatonin, a situation more analogous to daytime administration in the able-bodied and as such, the novel finding of a sedative effect in this situation is perhaps not surprising. 

An increase in morning and daytime sleepiness was reported by participants 1 and 4. This observation raises the possibility that chronic tetraplegia may be associated with an alteration in melatonin absorption, metabolism, and/or excretion. Such alteration may have resulted in higher circulating melatonin in these participants during the day and subsequently increased sleepiness. Further understanding and characterizing of the time course of melatonin concentration in people with tetraplegia is required. The determination of Cmax and the half-life of melatonin would help distinguish the delivery time of melatonin for this population. 

### 4.1. Limitations

The use of benzodiazepines, muscle relaxants, or breathing aids is often avoided in sleep research to limit any confounding effects they have on sleep architecture and quality. Unlike the able-bodied population, the prevalence of OSA and other symptoms resulting from the cervical spinal cord injury such as pain and spasm is extremely high [25]. Subsequently the prescription of these medications is very common. The continued use of these medications by participants in this pilot study was allowed in attempt to minimize the effect that these confounding symptoms have on sleep. Further, to not include people who had a diagnosis of OSA and to exclude those on treatment would severely limit the portion of the tetraplegia population which these results could be generalised to.

The scope of this study did give rise to limitations which future research should consider. This pilot study is limited by the number of saliva samples collected and sleep studies performed. An increase in sampling would not only provide additional, more complete information about the circadian rhythm of melatonin production in people with tetraplegia but would also control for possible night-to-night variability between baseline and postsupplementation testing sessions. Measures of other circadian markers, such as core body temperature, would assist in understanding the effect that melatonin supplementation has on circadian phase in this population. 

The nature of the home based setting in this study also gives rise to external variables which could not be controlled. The home based studies were selected for this study due to the logistic difficulties experienced in performing laboratory based studies in this population. Although the study presents data collected in the participants' natural living environment where sleep characteristics are not controlled by laboratory assessment and settings, it is difficult to monitor confounding variables such as diet and temperature. More obviously, this study is also limited by its sample size, which should be increased to validate the trends observed in this study.

This study showed that supplementation of 3 mg melatonin had an effect on both objective and subjective sleep for a person with complete tetraplegia. The improvement in subjective sleep experience suggests that melatonin supplementation may have a therapeutic role in tetraplegia. Due to the recruited sample size, however, only trends could be observed in this study with many changes following supplementation not reaching statistical significance likely due to a lack of statistical power. Therefore a larger, placebo-controlled, randomised trial is required to validate these findings. It would be of benefit to further explore melatonin absorption, the changes in sleep architecture, the apparent hypnotic effects, and the relationships between symptoms and sleep architecture that 3 mg melatonin supplementation has on people with SCI. 

## Figures and Tables

**Table 1:** Individual characteristics of each participant.

Participant code	Sex	Age (yrs)	BMI (kg/m^2^)	Injury level and AIS score	Years since SCI	Medications	Treatment
1	M	46	32.9	C5A	23.3	Baclofen, Temazepam	CPAP
2	M	68	27.7	C4A	23.9	Baclofen	CPAP
3	F	28	20.3	C5A	4.10	Baclofen, Diazepam	—
4	M	42	20.8	C6A	17.10	Baclofen, Diazepam	Oral Splint
5	M	26	18.5	C6A	10.10	Baclofen	—

Positive control							

1	M	43	24.8	—	—	—	—
2	F	31	20.7	—	—	—	—

BMI: Body Mass Index, AIS: American Spinal Injury Association Impairment Scale, SCI: Spinal Cord Injury, CPAP: Continuous Positive Airway Pressure.

**Table 2:** Sleep study findings recorded at baseline (the night before commencing melatonin supplementation) and on last night of two-week melatonin supplementation period.

	Baseline	Melatonin Supplementation
AI	6.2 (3.8–22.5)	5.8 (3.3–33.5)
AHI	7.6 (3.6–57.7)	3.5 (1.2–48.6)
Stage 1%	1.3 (0–4.4)	2.2 (1.7–2.9)
Stage 2%	45.9 (32.7–68.7)	61.3 (40–68.5)
Stage 3%	15.8 (4.1–35.1)	19 (3.4–32.3)
Stage 4%	14.6 (0–30)	10.9 (0–13.4)
Stage REM%	13.1 (7.8–36.1)	14.2 (10.8–25.2)
REM latency	92 (63.5–169.5)	152.5 (66.5–213.5)
TST	332.5 (90–353.5)	338.5 (292–365)
SpO2 < 90%	0.6 (0–37.6)	0.8 (0.4–21.1)
# Awake times	7 (4–24)	7 (4–40)
Time spent awake	16 (6–149)	20 (5–136.5)

Note: values are median (range). AI (Arousal Index) = number of arousals per hour of sleep; AHI (Apnoea/Hypopnoea Index) = number of apnoeas/hypopnoeas per hour of sleep; TST (Total Sleep Time) = minutes sleep; Time spent awake = minutes spent awake during night after first falling asleep. No statistically significant differences were observed between the baseline and melatonin supplementation sleep study parameters.

**Table 3:** Subjective sleep reports the night prior to (baseline) and last night of the two-week melatonin supplementation phase.

	Baseline	Supplementation
KSS	2 (2–4)	2 (1–4)
BNSQ1. Have you had any difficulties in falling asleep?	2 (1–5)	1 (1–3)
BNSQ2. For how long (how many minutes on average) do you stay awake in bed before you fall asleep (after lights out)?		
(a) Working days	5.8 (1.5–10)	5.5 (1–10)
(b) During free time	20 (1.5–60)	15 (1–30)
BNSQ3. How often do you wake up during the night?	5 (4-5)	5 (3–5)
BNSQ4. How many times do you usually wake up in one night?^b^	3 (3-4)	2 (2-3)*
BNSQ5. How often have you awakened very early in the morning without being able to fall back to sleep?	2 (1–3)	1 (1–4)
BNSQ6. How well have you been sleeping?^c^	3 (2–4)	2 (1–3)
BNSQ7. Have you used sleeping pills (by prescription)?	1 (1–5)	1 (1–5)
BNSQ8. Do you feel excessively sleepy in the morning?	1 (1–3)	3 (1–4)
BNSQ9. Do you feel excessively sleepy in the daytime?	1 (1–4)	2 (1–3)
BNSQ10. Have you suffered from an irresistible tendency to fall asleep while at work?	2 (1–3)	2 (1-2)
BNSQ11. Have you suffered from an irresistible tendency to fall asleep during free time?	2 (1–3)	1 (1-2)
BNSQ12. How many hours do you usually sleep per night?	6 (6-7)	6.5 (6-7)
BNSQ13. At what time do you usually go to bed (in order to sleep)?		
(a) During a working week	10:15 pm (9 pm–11:30 pm)	10:50 pm (9:40 pm–12 am)
(b) During free days	11 pm (9 pm–1:30 am)	12 am (9:40 pm–12 am)
BNSQ14. At what time do you usually wake up?		
(a) During a working week	6:30 am	6:30 am
(b) During free days	6:30 am (6:15 am–8 am)	6:30 am (2:30 am–9:30 am)
BNSQ15a. How often do you have a nap during daytime?	1 (1–4)	1 (1–3)
BNSQ15b. If you take a nap, how long does it usually last (minutes)?	40 (20–60)	32.5 (20–45)
BNSQ16. Do you snore while sleeping (ask other people)?	2 (1–3)	3 (1–5)
BNSQ17. In what way do you snore (ask other people about the quality of your snoring)?^d^	3 (1–5)	3 (1–5)
BNSQ18. Have you had breathing pauses (sleep apnoea) during sleep (have other people noticed that you have pauses in respiration when you sleep)?	1	3 (1–5)
BNSQ20. How many hours of sleep do you need per night (how many hours would you sleep if you had the possibility to sleep as long as you need to)?	7 (6–10)	7 (6–10)

*P < 0.05. Note: values are medians (range). The basic scale for answer alternatives is as follows: 1 = never or less than once a month; 2 = less than once per week; 3 = on 1-2 days per week; 4 = on 3–5 days per week; 5 = daily or almost daily.^b^ Answer alternatives: 1 = usually I don't wake up at night; 2 = once per night; 3 = two times; 4 = 3-4 times; 5 = at least five times per night^c^. Answer alternatives: 1 = well; 2 = rather well; 3 = neither well nor badly; 4 = rather badly; 5 = badly^d^. Answer alternatives: 1 = i don't snore; 2 = my snoring sounds regular and it is of low voice; 3 = it sounds regular but rather loud; 4 = it sounds regular but it is very loud (other people hear my snoring in the next room); 5 = i snore very loud and intermittently (there are silent breathing pauses when snoring is not heard and at times very loud snorts with gasping).
